# Haiti’s HIV Surveillance System: Past, Present, and Future

**DOI:** 10.4269/ajtmh.20-0004

**Published:** 2020-07-20

**Authors:** Chris Delcher, Ermane G. Robin, Daniella Myriam Pierre

**Affiliations:** 1 College of Pharmacy, University of Kentucky, Lexington, Kentucky;; 2 Programme National de Lutte contre les IST/VIH/SIDA (PNLS) Unite de Coordination des Maladies Transmissibles (UCMIT) Ministere Sante Publique et Popuation (MSPP), Port-au-Prince, Haiti

## INTRODUCTION

In Haiti, HIV epidemiology indicates that in 2018, 160,000 people were living with HIV, 7,300 people were newly infected with HIV, and 2,700 people died from an AIDS-related illness (seventh leading cause of death in Haiti). Among people living with HIV (PLWH), only two-thirds knew their HIV status and just 58% were receiving treatment.^1,2^ One cornerstone of HIV surveillance in Haiti was the implementation of the national HIV case-based surveillance (CBS) system known as Suivi Actif Longitudinal du VIH en Haiti (SALVH).^3^ The purpose of this article was to describe SALVH’s development, key contributions to population health, emerging operational threats, and future directions.

## CASE-BASED SURVEILLANCE

According to the U.S. Centers for Disease Control and Prevention (CDC), CBS is the “systematic reporting of newly diagnosed (PLWH) at the time of diagnosis and if applicable subsequent reporting of key clinical events (sentinel events) for that individual during the course of the disease. Case-based surveillance data are individual-level data that is de-duplicated across healthcare facilities and over time using a set of identifiable variables or a unique identifier.”^4^ The Joint United Nations Program on HIV/AIDS (UNAIDS) and the World Health Organization (WHO) have enumerated five components of integrated HIV “second-generation surveillance” systems inclusive of CBS: 1) HIV case morbidity and mortality reporting (i.e., CBS), 2) sexually transmitted infection surveillance, 3) biological and behavioral surveillance of key populations, 4) sentinel surveillance of select populations, and 5) size estimation of key populations.^5^ Case-based surveillance, the focus in the following sections, is one part of a multipronged HIV surveillance strategy that should triangulate with the components listed previously.^6^

## DEVELOPMENT OF CBS IN HAITI

In 2003, UNAIDS estimated Haiti’s adult HIV prevalence at 5.6% (highest in the Western Hemisphere) with ∼280,000 PLWH.^1,7^ Around that time, prevalence rates among pregnant women were > 1%, thus qualifying Haiti’s epidemic as “generalized” by the WHO standards.^8^ In turn, Haiti was designated a priority country for the U.S. President’s Emergency Plan for AIDS Relief (PEPFAR) support to the national HIV response, and the Haitian government rapidly expanded HIV prevention and care services by establishing a national network of facilities for voluntary testing and counseling, prevention of mother-to-child transmission, antiretroviral treatment (ART), and palliative care.^9,10^ Simultaneously, a CBS system^11^ (aka, sentinel or event-based surveillance^12^) was established to assess incident/prevalent HIV infections with an emphasis on identifying distinct patient records. The latter feature avoids the limitations of using aggregate counts of monitoring and evaluation data and enables longitudinal patient monitoring.^13,14^

In 2008, CBS from HIV testing and care clinics was established by electronically transferring data from Haiti’s three largest clinical networks.^3^ Rapid scale-up was possible because Haiti had a manageable number of electronic medical record systems covering most PLWH, strong political/organizational support, and local technical/programming expertise.^15–18^ Suivi Actif Longitudinal du VIH en Haiti’s development cycle may not be achievable in some contexts; CBS systems are still rare in low- and middle-income countries. For example, according to the PEPFAR HIV CBS Study Group, among 39 PEPFAR countries, only 20 (Haiti included) had reported implementing CBS by 2019.^4^

Suivi Actif Longitudinal du VIH en Haiti expanded over the next decade (even recovering from the massive earthquake of January 12, 2010^19^); tracked events now include clinical progression markers (e.g., CD4 counts), healthcare visit dates, and ART dispensing dates.^20^ Thus, SALVH provides the Haitian government the ability to examine clinically relevant outcomes such as linkage to care, retention in care, access to treatment, viral load suppression, and patient transfers for key populations (e.g., pregnant women, sex workers, and men who have sex with men).^21,22^ A few illustrative examples on how SALVH has delivered on the vision to improve population health are provided in the following sections.

## GLOBAL SUCCESS

Fundamentally, HIV CBS should provide accurate counts of PLWH. Haiti lacks a ubiquitous national unique person identifier, so an approach to de-duplication was designed and validated, finding that national estimates of PLWH would have been 30% higher without accounting for same-patient visits erroneously presumed to be different-patient visits.^3^ Simultaneously, this SALVH feature provides insight on patients presumed lost to follow-up (LTFU), as a large proportion of these patients transfer to a different clinic. We found that these “silent transfers” occurred more frequently during a period of rapid scale-up of HIV clinics in Haiti.^23^ In another study, researchers found ∼10% fewer patients were LTFU because they used SALVH to identify them visiting other clinics.^24^ Anecdotally, SALVH’s downward patient count adjustments assuaged concerns that patients were frequently visiting multiple clinics inappropriately (i.e., “medical shopping”).

Suivi Actif Longitudinal du VIH en Haiti’s sentinel event tracking has supported several key studies. One study showed that Haitian PLWH initiating ART had higher CD4 counts (i.e., they were healthier) than PLWH in nine comparator countries.^25^ Haiti’s participation in that study, especially one highlighting a success rather than a failure of its public health system, is testament to SALVH and its partners. Recently, a SALVH data team was organized to support the “clinical cascade” approach.^26,27^ This research team, referred to as the “Groupe d’Analyses SALVH” and inclusive of multiple Haitian colleagues from private and public sectors, published a series of HIV care and treatment cascades using SALVH data.^28^ Interestingly, just one year before this study, a systematic review of the clinical cascades in 69 countries was completed.^29^ Unfortunately, Haiti missed inclusion, as the Groupe d’Analyses SALVH was still working to make these cascades available.

## HOW DOES CBS CONTRIBUTE TO CARE CASCADES?

Recently, the CDC reviewed CBS systems in 39 PEPFAR-funded countries.^4^ Here, we informally examined whether reporting out a national clinical cascade (complete or partial) was associated with CBS status (implementing or planning). Among the 12 countries with CBS implemented, seven countries (Brazil, Nicaragua, Rwanda, Thailand, Ukraine, Vietnam, and Zimbabwe) had complete clinical cascades and five countries (Botswana, Guatemala, Jamaica, Panama, and Senegal) had partial cascades. Among the nine countries with CBS planned, eight countries (Côte d’Ivoire, Malawi, Mozambique, Nigeria, South Africa, Tanzania, Uganda, and Zambia) had partial cascade data available. Thus, CBS does not guarantee comprehensive data, but countries in CBS planning-only stages mostly lacked complete cascades (except Kenya). Thus, we hypothesize that CBS is critical for the complete picture of an HIV continuum of care that cascades are intended to provide. The CBS status was self-reported and lags the cascade data by several years.

## LOCAL SUCCESS

The aforementioned examples are the most visible of SALVH outputs, but how is SALVH used in Haiti? SALVH data contribute substantially to HIV epidemiology profile reporting by the Ministry of Health and Population known as Ministère de la Santé Publique et de la Population (MSPP). To demonstrate, we examined publicly available epidemiologic surveillance bulletins and statistical and other reports published by MSPP^30^ (2013 forward) and found 169 instances of SALVH-generated data and/or references. Suivi Actif Longitudinal du VIH en Haiti has become a central component of Haiti’s health information strategy. The system provides data linkages to other complementary health information systems such as active perinatal surveillance (Surveillance Active de la Femme Enceinte Seropositive) to monitor visits that help prevent mother-to-child transmission and an outreach program to identify patients presumed LTFU by sending health workers to find them in their home communities. Collaborating with MSPP, SALVH data dashboards were developed using Google’s Data Studio platform^31^ to automate routine reporting functions and expand the SALVH user base.

## NEW THREATS TO STAFFING

Despite these successes, rapid funding cuts could threaten Haiti’s ability to maintain CBS and SALVH’s infrastructure which includes software, hardware, and staffing components. The United States has proposed reductions in current funding levels (approximately 30% to both PEPFAR and the Global Fund to Fight AIDS, Tuberculosis, and Malaria).^32,33^ The National HIV Control Program in Haiti, the agency now overseeing SALVH staff, budget was cut by $7 million, and one key U.S. partner maintaining SALVH’s workforce, the National Alliance of State and Territorial AIDS Directors, has closed local offices. Some SALVH staff were successfully transferred, others found alternative work, and the data infrastructure is now managed by a local private IT partner. But, what happens if these deep funding cuts threaten SALVH’s operations? We searched the scientific literature to find examples of outcomes following the loss of a mature CBS; to our knowledge, none exists. In Haiti, the national picture of HIV epidemiology would certainly blur and population health monitoring activities mentioned earlier diminished. Data flow could potentially be maintained (because of efforts to automate data transfer), but in the absence of trained and diligent public health analysts, SALVH would not yield actionable information. The scope here is too limited to imagine all possible outcomes, but just consider the example of SALVH staffing interruptions that could delay the patient record matching process. As a result, national HIV case counts could artificially increase, which may falsely be interpreted as a significant HIV outbreak. The expertise required to interpret sudden changes in the data is not easily replaced. An astute staff member once determined that a single-month increase in HIV reporting occurred at precisely the time the U.S. ambassador to Haiti visited, likely the result of a hurried attempt to reduce a data entry backlog.

## NEW DIRECTIONS

Advanced methods for patient record matching (e.g., machine learning) and enhanced data elements (e.g., phone numbers and biometrics) to improve automatic record identification have also been proposed. For example, in 2016, MSPP introduced fingerprinting in 160 healthcare clinics providing HIV services. An analysis of SALVH’s existing algorithm method compared with fingerprint matching showed a 6% reduction in counts of PLWH by reducing false-positive matches (26,988 unique PLWH without fingerprint matching versus 25,154 unique PLWH with fingerprint matching).^34^ These technologies have benefits beyond counting cases (e.g., finding patients lost to follow-up), and to the extent that these technologies are culturally appropriate, use and validation should be encouraged.

## CONCLUSION

Suivi Actif Longitudinal du VIH en Haiti is promoted as a success story in the WHO’s guidance for CBS around the world.^35^ We remain optimistic that, by providing evidence of success and challenges, SALVH will continue to exemplify CBS during this vulnerable time. At a minimum, we hope that this perspective adds to the historical record of CBS in Haiti. This year Haiti commemorates the anniversary of the January 12, 2010 earthquake, one of the largest human catastrophes in recent memory. In retrospect, one of the positive outcomes of the tragic earthquake was the influx of resources that sustained SALVH which, as we have argued here, has provided a significant public health return for the people of Haiti. Moving forward, we need only remember the nature of the word *surveillance*—to keep watch over—to appreciate that CBS requires sustained investment and oversight.
